# *‘There is nowhere to take the child’:* a qualitative study of community members’ views on managing early childhood substance use in Mbale, Uganda

**DOI:** 10.1186/s12889-022-13548-4

**Published:** 2022-06-15

**Authors:** V. Skylstad, I. M. S. Engebretsen, S. J. Nalugya, C. Opesen, G. Ndeezi, E. S. Okello, K. M. Moland, J. K. Tumwine, A. M. S. Skar

**Affiliations:** 1 Centre for International Health, Department of Global Public Health and Primary Care, Faculty of Medicine, University of Bergen, Bergen, Norway; 2 Department of Psychiatry, School of Medicine, Makerere University College of Health Sciences, Kampala, Uganda; 3 Department of Psychiatry, Mulago National Referral and Teaching Hospital, Ministry of Health, Kampala, Uganda; 4 Department of Sociology and Anthropology, School of Social Sciences, Makerere University, Kampala, Uganda; 5 Department of Paediatrics and Child Health, Makerere University College of Health Sciences, Kampala, Uganda; 6 Mwanza Intervention Trials Unit, National Institute for Medical Research, Mwanza Campus, Mwanza, Tanzania; 7 Department of Paediatrics and Child Health, Kabale University School of Medicine, Kabale, Uganda; 8 Global Health Cluster, Norwegian Institute of Public Health, Oslo, Norway; 9 Norwegian Centre for Violence and Traumatic Stress Studies, Oslo, Norway

**Keywords:** Childhood substance use, Community health response, Social determinants of health

## Abstract

**Background:**

Harmful alcohol use by 5–8-year-old children has been identified in Mbale District, Uganda. To further examine this finding, the present study explores the experiences and perceptions of community members regarding how childhood substance use (before age 10) is managed in this area.

**Methods:**

We conducted eight focus group discussions with 48 parents of children aged < 10 years and 26 key informant interviews with teachers, health workers, child protection workers, police, local stakeholders, brewers, and others. Thematic content analysis was performed.

**Results:**

Three main themes were identified:

*‘We don’t talk about it’:* Despite concern, childhood substance use was not addressed in the community. Participants attributed this to three main factors related to a lack of leadership in addressing it, changing acceptability for peer parental interference, and uncertainty about repercussions related to children’s rights.

*‘There is nowhere to take the child’:* Schools, police, and remand homes were intuitively considered appropriate arenas for managing childhood substance use but were considered inaccessible, unresponsive, and inadequate due to insufficient resources, competence, and training. Since substance use was not considered a medical problem, help from the health sector was only sought for adverse consequences, such as injury. This left the participants with the experience that there was in effect nowhere to take the child.

*‘The government has not done so much’:* The participants called for government action and clear laws that would regulate the availability of alcohol and other substances to children, but they had limited trust in the capacity and commitment of the government to act.

**Conclusions:**

The participants were concerned about childhood alcohol and substance use, but the complexity and magnitude of the problem left them feeling incapacitated in responding. Relevant factors were identified on the community, institutional, and the government level, such as a lack of leadership in addressing it, a loss of mandate to interfere in child-rearing, inadequate services, weak legal structures, and missing government action. A strengthening of collective agency and public policy is necessary to prevent and address childhood alcohol and substance use.

**Supplementary Information:**

The online version contains supplementary material available at 10.1186/s12889-022-13548-4.

## Background

There is ample evidence that substance use and dependence have health related, economic, and social consequences for individuals and communities globally [1, 2]. The patterns of substance use vary across the globe, but alcohol dependence is the most common substance use disorder in most countries [1]. The World Health Organization (WHO) states that “*the harmful use of alcohol compromises both individual and social development. It can ruin the lives of individuals, devastate families, and damage the fabric of communities.*” [3]. According to the 2018 WHO Global Status Report on Alcohol and Health, the WHO African region had a level of alcohol per capita consumption (APC) similar to the world average, at 6.3 L. However, the region had a pattern of high abstention rates and high APC among drinkers and the highest age-standardised alcohol-attributable burden of disease and injury [2]. Uganda, once ranked with the world’s highest APC in 2004 [4], had an APC of 9.5 L in 2016, almost 50% higher than the African region average [2]. Among men, the APC was 16.1 L and among women it was 3.0 L [2].

The WHO Global Strategy to Reduce the Harmful Use of Alcohol emphasises community action as an important target area for interventions to prevent and respond to harmful alcohol use [3]. To plan appropriate preventive and treatment interventions, it is instrumental to understand how context-specific practices, attitudes, and culture influence the community handling of mental illness and substance use [5]. There are long-standing traditions for brewing alcohol in Uganda [6], and 86% of their consumption comprises of unregulated local brews, such as fermented beverages made of banana and grains [3]. While men drink more, and their intake is more socially accepted [6], women are the primary brewers. This practice has been linked to their children being exposed to brew in the process of brewing and selling [6, 7].

Alcohol and substance use can be especially detrimental when it starts early in life [1, 8, 9]. Data from the Global School-Based Student Health Survey show that alcohol intake before age 10 or 11 ranged from 4.1–43.5% between 45 low- and middle-income countries (LMIC) [10]. Compared to later onset, pre- and early adolescent alcohol intake has shown a stronger association with later life dependence [11], lower scores on the Social and Occupational Functioning Assessment Scale [12], and poorer psychomotor speed, visual attention, working memory and cognitive inhibition [13]. The mechanisms include biological explanations, where alcohol directly affect the developing brain and cognitive skills [14], but also play into the complex interactions with social determinants of health, including poor education outcomes and early pregnancy [15, 16].

In 2014, we identified harmful alcohol use in a small sample (*n* = 148) of 5–8-year-old children living under parental care in Mbale District, eastern Uganda [17]. This finding spurred the present study, aiming to further explore and understand early childhood substance use, defined as before age 10 years. We consider ‘substance use’ to be the intake of any psychoactive substance, including alcohol. In the context of Mbale, alcohol was the most common substance of use, and was the main substance discussed by the participants. However, other substances were also relevant, such as cannabis, kath, kuber and solvents for sniffing, such as fuel or glue. In a previous paper from this study (i.e., the same participants and dataset) we described the social determinants related to the context and conditions for early childhood substance use, finding that alcoholic brews and other substances were widely used in daily life and ceremonies from the first year of life, but the use was exacerbated by deprivation and exposure to stressful and traumatic experiences [18]. Children of poor families, brewers, slum dwellers, internally displaced people and street-connected children were considered particularly vulnerable for using brew and other substances to cope with traumatic events, hunger, and neglect [18].

Despite being a leading cause of the disease burden among youth [8, 9], an analysis of data from the World Mental Health Surveys showed that 99% of patients of all ages with past-year substance use disorders in LMICs remained without minimally adequate treatment [19]. According to data from the WHO Global Health Observatory repository, there is no epidemiological data collection system for substance use among children and adolescents and no treatment programmes for children and adolescents with alcohol or substance use disorders in Uganda [20]. The treatment gap in child and adolescent mental health services in LMICs has been connected to family and community factors, as well as structural, political, psychosocial, and sociodemographic factors, service delivery and inadequate reach of vulnerable populations [21, 22].

The current paper explores how early childhood substance and alcohol use is addressed and managed at the community, institutional and government level, and factors that influence agency. Our discussion is underpinned by the WHO Commission on Social Determinants of Health framework on social determinants of health, focusing on elements related to community factors, governance, power, social cohesion, and the health system [22]. We use this framework because it allows us to consider the complexity of the wider social contexts of early childhood alcohol and substance use, as opposed to specific pathways for deviant behaviour.

## Methods

### Study design and setting

We applied a qualitative study design, combining key informant interviews (KIIs) and focus group discussions (FGDs).

The study was conducted from April-June 2016 in the Mbale District in eastern Uganda. In the last census from 2014, Uganda’s population counted approximately 35 million [23], but according to United Nations data the total population in 2022 has been estimated to be 48.5 million [24]. Thus, the country is experiencing a rapid populations growth, and with 50% below 15 years, it has one of the world’s youngest populations [23]. According to the 2014 census, 500 000 people were living in Mbale District and 95 000 were living in the urban centre of Mbale City [23]. The district lies in a tropical area around the foot of Mount Elgon. Large lines of transportation of goods run through Mbale as it has road connections to large cities within Uganda, and to the Kenyan border in the east. Several ethnic groups reside in Mbale, including the Bamasaba originally from the area, Banyole, Bagwere from surrounding eastern areas, Baganda from the central region, and Iteso and Karamojong from north-eastern areas. The main languages are Lumasaaba, Luganda and English. The social indicators vary greatly within the district: 8.2–29.3% (average 13.9%) of children aged 6–12 years were not in school, 2.6–15.5% (average 9.6%) of households consumed less than two meals a day, and 1.2–57% (average 14.7%) lived 5 km or more from a public health facility [23]. There is one main urban centre, Mbale City, which holds approximately 20% of the district population, and hosts the main district referral hospital that has a psychiatric ward.

### Participants and sampling

We conducted eight FGDs, for which we purposively sampled six parents of children younger than 10 years to ensure they had a clear understanding of the age group. We did not collect data on the number of children they had or their ages but, considering the age range of the participants (18–76 years, see Table 1), we anticipate that their children spanned all ages below age 10. To enhance group dynamics, we sought homogeneity within the groups relating to gender (male or female) and age (18–30 years or 31 years and older), while seeking heterogeneity between the groups, anticipating that this would enable representation of different perspectives. We therefore included participant groups from different communities, i.e., urban/rural residency, slum areas, and agricultural areas. This selection was aided by the research assistants’ knowledge about the district and the aim of covering a wide range of residential and social backgrounds. Within the selected communities, a community mobiliser helped recruit relevant participants based on their prior knowledge about community members fitting the eligibility criteria. We did not collect information on how many were approached and declined to take part. None of the participants withdrew after inclusion. Only one FGD was conducted in each selected community.

**Table 1:** Participant characteristics, reproduced from Skylstad et al., 2022 [18]

Focus group discussion with parents		Key informant interviews	
	N		N
**Total**	48	**Total**	31
Female	24	Female	14
Younger age (mean: 24 years, range: 18–30)	30		
Older age (mean 49 years, range: 31–76)	18		
*Main occupation*		*Main occupation*	
Farmer	24	Primary school teacher	2
Student	6	Health worker	5
Trader	5	Youth worker	5
Craftsperson	4	Lawyer	1
House wife	2	Police officer	1
Local chairman	2	Mental health activist	2
Qualified professional	2	Religious leader	1
None	1	Alcohol distributor	3
No answer	2	Pharmacist	1
*Education level*		Community stakeholder for children	8
Primary (P1-P7) only	21	Government official	1
Secondary (S1-S6) only	20	Traditional healer	1
High school, A level	1		
Tertiary degree	3		
No formal education	1		
No answer	2		

The KIIs were done in two ways, 24 individual interviews and two group interviews with three and four participants from the same organisation. The group interviews were organised on the initiative of the organisation, as more representatives wished to share their insights and experiences. Appreciating their initiative and interest in taking part, we accepted to include more participants in the same interview. We purposively sampled participants we believed would have relevant information by using our network, visiting relevant institutions and snowball sampling, where participants recommended other participants. Participants in these interviews included teachers, community leaders, youth workers, police, religious leaders, health workers, mental health activists, a pharmacist, child protection workers, traditional healers, and alcohol distributors. All the approached participants accepted to take part in the study.

### Process and procedures

The FGDs were held in the participants’ home community. Two research assistants facilitated the FGDs. Both were female, experienced with qualitative research, fluent in the local languages, and held a bachelor’s degree in social sciences and community psychology, respectively. While one moderated the discussion, the other observed and took notes. The KIIs were primarily held by the first author (see ‘Reflexivity’ section for details), in a location chosen by the participants.

The participants were given oral and written information about the study. We used a topic guide to structure the interviews and discussions. The focus group discussions started with the facilitator reading a vignette about a boy and a girl using alcohol before age 10 (see supplementary file 1). The purpose was to spur the discussion through a hypothetical case that presented a situation that the participants could recognise from their own communities. This was deemed a better entry point to a potentially sensitive topic than asking for the participants' personal experiences in a group setting [25]. The vignette was developed based on observations in the community. The topic guide was tried out within the research team and was inductively modified during data collection to capture new and relevant topics. The participants shared openly about their experiences and perceptions and did not seem hampered by the sensitive nature of the topic in either the FGDs or KIIs.

The FGDs and three KIIs (two with alcohol distributors and one with a traditional healer) were held in the Lumasaaba language and transcribed directly into English by the two research assistants, who agreed on the translation. The first author conducted 23 of the KIIs in English and transcribed the interviews verbatim. All FGDs and KIIs were audio recorded and lasted between 60–120 min (average 80 min). During data collection, the first author and research assistants discussed each transcript, the need for further probing and clarifications of the content. Data collection was continued until saturation was deemed met upon agreement that no new themes seemed to arise, and a broad variety of participants was represented.

### Analysis and interpretation

The unit of analysis comprised the transcripts of KIIs and FGDs. These were analysed as one dataset, since our intention was not to compare findings from different research methods or categories of participants (i.e., gender, ages, place of residence), but to reach a nuanced and comprehensive exploration of perspectives on a complex topic. The transcripts were read and reread to gain a sense of the whole before and after thematic content analysis, to ensure representativeness of the findings [25]. The first impressions were discussed with a Ugandan medical anthropologist (ESO). The first author read the full unit of analysis, while IMSE and AMSS read a selection of transcripts to be familiar with the data. The first author coded the transcripts using NVIVO 12 and sorted codes in Office Word to identify categories and themes, which were iteratively amended throughout the process of analysis and writing. Further, the analysis was implicitly informed by observations and field notes, considering whether the data matched observations in the field. We used an empirical data-driven and inductive approach. The codes and themes were discussed within the team, and representativity was verified by rereading full transcripts. We selected quotes to illustrate the theme, aiming for wide representation of participants. For context, we labelled the quotes with the role for which the participants were purposively sampled, while ensuring their anonymity. Identified themes that related to the context and conditions (i.e., culture, family conditions, poverty and traumatic experiences) for childhood alcohol and substance use has been presented elsewhere [18].

### Reflexivity

The first author is a Norwegian female with experience and special interest in addiction medicine. She was a medical student with experience in qualitative research at the time of data collection and a medical doctor and PhD candidate at the time of analysis. Though she had spent cumulatively one year in Uganda, her position may have impacted the interaction with participants and the data. To explore this, the first impressions were discussed with Ugandan colleagues, including a medical anthropologist (ESO). The general impression was that the outsider position yielded rich descriptions, where the participants assumed her limitations in knowledge. They did not seem intimidated or constrained in their sharing. Further, the information from the FGDs and KIIs where the first author was not present﻿ was largely overlapping with the information from the KIIs led by the first author. Our impression was that the rich and open sharing in both the KIIs and FGDs had allowed for viewpoints beyond socially desirable answers, and there was a clear impression of agreement across participant gender, age, and sociodemographic backgrounds. The topic engaged the participants, and many expressed their gratitude for the opportunity to share. However, appreciating the complex social mechanisms at play in both interviews and groups discussion, and the limitations they may pose to reach a complete and true representation [25], participant validation of the findings was sought. A draft of the results was shared with participants from the KIIs that had consented to later be contacted for clarifications and analysis, and the research assistants were invited to provide feedback based on their impressions from the FGDs. Four participants from the KIIs and one research assistant responded, emphasising their agreement with the results as presented. None wished to make any amendments.

## Results

We identified three main themes related to handling childhood substance use in Mbale, Uganda. In the first theme *‘We don’t talk about it’*, participants explained challenges related to leadership in addressing the problem and a lack of mandate to intervene with other parents’ children. In the second theme *‘There is nowhere to take the child’* we explored the perceived relevance and ability of the school, legal system, and health sector to manage childhood substance use. In the third theme, *‘The government has not done so much’* we found that government involvement was perceived necessary, but the trust in their commitment and ability to act was limited. Further, throughout the themes we explored the factors within and the interplay between community action, relevant institutions, and governance. We used the term ‘substance’ for any psychoactive substance, including alcohol, and we named the substance when appropriate.

### Addressing childhood substance use: ‘We don’t talk about it’

The participants were deeply concerned about childhood substance use. Despite this, it was not part of community discussions, primarily explained by three factors: 1) a lack of leadership in addressing it, 2) changing acceptability for peer parental interference and 3) uncertainty about repercussions related to children’s rights. Bringing up the topic was perceived difficult, requiring leadership. Further, the community members’ mandate to intervene and guide other parents and their children was curtailed by changes in the social fabric of the community, where child-rearing responsibilities were increasingly vested in the nuclear family. Children’s rights exacerbated their hesitancy to interfere, leaving participants scared of legal prosecution if they intervened using corporal punishment. Many participants expressed a wish to do something about early childhood substance use, if only they had the power and community mandate to raise it.

#### ‘It requires a leader’

The participants expressed a deep concern for childhood substance use and explained that community sensitisation was necessary. Simultaneously, they demonstrated that they, as community members, were well sensitised to the issue, and shared knowledge about harmful substance use and its consequences. Further, they explained that the existence of childhood substance use was indeed common knowledge but that it was not easy to address: “*Everyone knows about it! A majority of people know about it but coming out as a single individual to say that 'no this practice is wrong', it is not simple.” (KII 19, youth worker)*. Therefore, despite concern, there was a recurring notion of despair and powerlessness to handle the problem. A part of the challenge was that the practice of drinking alcohol was perceived to be so prevalent and accepted in the community that standing up against it was difficult for single individuals. While everyone knew, they were waiting for someone else to take initiative and leadership in bringing up the conversation.

“*There is no discussion about it because it [drinking] has become a habit. How do you start that [discussion] when bars are everywhere? We don’t talk about it. How do you start it? It requires a leader of the village. I cannot just tell someone to stop drinking, they may not listen, it requires a leader*.” *(FGD 8, younger men*).

Leadership was also considered necessary when approaching and addressing specific parents of children that used substances. Local council members and those with an official role in the community experienced that they had the authority and mandate to guide parents when necessary, and many participants pointed to them for action. For community parents to address other parents directly, however, was considered challenging in a context where parenting practices were changing.

#### ‘We have lost our original African vibe of parenting’

The lack of discussion in the community co-occurred and was influenced by a change in social community structures, particularly related to child-rearing. The participants experienced that while they were free to discuss and discipline children in the community in the past, the responsibility for raising children had moved from the collective community to the nuclear family. A police officer explained that “*We have lost our original African vibe of parenting” (KII 17, police officer).* This limited their mandate to intervene with and guide other parents and children, as interference from community members was increasingly unwelcome:

“*When we were young, they told us that whoever you would meet on the way, a woman is your mother, and a man is your father. That ‘parent’ was also allowed to cane you if you had done something wrong […] But nowadays children cannot behave, and we can’t discipline them because we don’t have the same authority that we used to have.*”* (FGD 1, older men).*

The participants explained that many children and parents would reject community members that tried to address a child’s behaviour, including substance use. In addition to having lost the authority to intervene, they had lost the insight into what happened in the neighbours’ home. They alluded to an increased distance between community members and reduced connectivity in the community, expressed by the noted unwanted interference with the nuclear family and erected fences around compounds. In addition to the change in the collective nature of child-rearing, the introduction of children’s rights exacerbated the hesitancy to interfere, leaving many uncertain about what they were legally allowed to do.

#### ‘We now have to handle children like glass’

Children’s rights and their legal implications were a major concern and barrier to addressing, discussing, and getting involved with other parents’ children. They explained that children would bring this up if they were approached by a community adult; “*Nowadays, you don’t talk about someone’s child because if you talk [to] the child he runs and says ‘what does the children’s rights say?’. People fear to talk about those children.” (FGD 3, younger men).* Many participants complained that children’s rights made corporal disciplining illegal, removing an important tool for correcting behaviour. This challenge was also reported by participants working to protect children’s rights, who agreed with the concept of the rights, but recognised that they had not been well received in the communities. A youth worker explained:

“*They will say 'no, long ago, there were no children rights, we grew up and we were very obedient’. In fact, they believe it is a white man’s thing, these 'children's rights, they say ‘why do you want to spoil [ruin] our children by telling them they have rights?'*”* (KII 19, youth worker).*

Although not true for everyone, many participants reported that these laws, sometimes in combination with hostile reactions from parents or children, made them hesitant about future attempts of intervention for children that use substances:

“*We now have to handle children like glass. If I catch a child [using substances] and cane him or her, the child may go straight to the police and report, and they imprison me just because I am helping to put the child on the right track. Such things threaten us [from intervening] and it has caused the children to get spoilt [undisciplined]. So, there is an obstacle on the side of the government and on the side of the parent there is also an obstacle. The lack of having a say about it hurts us.*”* (FGD 6, older men).*

Changes in interpersonal community relations were further complicated by uncertainties about legal consequences. This worked to silence the community members when facing childhood substance use.

### Managing childhood substance use: ‘There is nowhere to take the child’

The participants considered institutions within education, law, and health intuitively appropriate for helping children that used substances, but did not consider them to be real alternatives in practice. Schools, remand homes, police and local council members were considered especially relevant, but insufficient resources, competence and training made these institutions unresponsive and inadequate. Religious leaders and traditional healers were mentioned by some, and there was disagreement on the relevance of the formal health system. This left the participants with the experience that there was in effect nowhere to take the child.

#### ‘The majority are sent away from school’

The participants believed that the school sector and teachers should be involved and prepared to help children that use substances. However, some believed that it was currently relying on the engagement of the individual teacher:

“*I think the schools have no clear program and strategy for handling cases of alcoholism. Some teachers try to help these children, but the majority [of children] are sent away from school. They look at them as a bad influence. You will just be lucky when the teacher is attending to the child.*”* (KII 4, youth worker).*

Many participants believed that the inaction was related to a lack of resources in the schools, while some believed the teachers lacked insight into the complexity of the issue and how to best understand and handle these children:

“*Some teachers mishandle children who are addicts, making some of them drop out. They don’t listen to the problems of these children. The teachers should be empowered and maybe given some capacity training on how to handle children in such families, or who have friends who are already addicts. They should also be trained on that.*”* (KII 1, primary school teacher).*

While participants believed the schools should and could be an important place to turn, the missing guidance on how teachers should understand and respond to childhood substance use made their reactions unpredictable.

#### ‘The police has failed’

Correctional interventions, such as the police and remand homes, were frequently discussed. Participants argued that the police should be involved to arrest the child or parent, and to deal with the violence and crime that substance use was associated with. Some participants reported a functioning collaboration between the local council members and the police, while many felt the police managed the problem inadequately:

“*I want to put in place a law so that the government can help us control children drinking. Here, children have defeated us and have even scared the police. We have so many who take marijuana and they have stones [are violent], and the police has failed.*”* (FGD 2, older women).*

This view was shared by a representative of the police, who explained that the problem was far bigger than the resources to tackle it:

“*I see this everyday [...] I see them sniff, see them smoke, see them chew. I used to arrest them, and the matter has come up several times in our district security meetings. Children are not criminally responsible, so […] they are remanded in the children's remand home here, which has a capacity of 43 children for 11 districts. In a single operation, I can arrest more than 100 children. So, what do you do? I see them, but what do I do. Tracking the parents down, arresting them and bring them to court is costly. We may not have the funds.*”* (KII 17, police officer).*

The lack of resources and the barrier of high costs were also felt at the individual level, where having a child in a remand home incurred impossible costs, leaving this option inaccessible in practice:

“*P1: We had a remand home for children, but nowadays you have to pay 450,000 Uganda shillings [approx. 120 US dollars]. So, there is no way you can shape them. P2: There is nowhere to take the child. P1: If you take him there, you will pay money every month and sometimes you are too poor to afford the money they are asking for, and the child ends up getting spoilt [undisciplined].*”* (FGD 1, older men).*

Further, the remand homes had a lower age limit of 12 years, leaving the institution unavailable for those below age 10. While police and remand homes were in theory considered to be part of the solution, both community members and the police recognised that there was no use in contacting them. Even in the unlikely event of getting access to an overburdened remand home, the parents would not be able to sustain the cost.

#### ‘Here in the hospital, we don’t see them’

There were varying perceptions about the relevance of the formal health system for managing childhood substance use. Some of the participants who were health workers shared their experiences with treating children as young as 10–13 years for withdrawal symptoms from alcohol, implying that the use had started earlier. However, this was rare since addiction was not generally perceived to be a medical problem, unless it had led to other consequences:

“*I know children below 10 years who drink, although here in the hospital we don't see them, but in the communities, they drink because they are brewing […] Those who get a problem because of drinking, a medical problem, a physical problem, or a mental problem, that is when they go to any health centre. If it was serious, they are brought to psychiatric care. People don't know that substance use is a medical problem.*”* (KII 16, health worker).*

Others pointed to barriers, such as high costs, stigma, and unfriendly health personnel. Others again believed that the health workers' understanding of substance use did not resonate with the understanding of the community members:

“*We in the medical field fail to eradicate some conditions because we want to separate the cultural from the disease, and it is wrong. To help solve the problem of alcohol abuse in children, and you know it is so culturally deep rooted, we should join them and modify the cultural practices, but we are not going to uproot the culture practice.*”* (KII 20, health worker)*.

In essence, except for a few cases, participants explained that children did not receive help from the health system. Challenges related to differences in the understanding of substance use, its medical relevance and barriers related to access and unpleasant experiences left opportunities for management and follow-up within the health system untried.

### Action for childhood substance use: ‘The government has not done so much’

The participants agreed that government initiatives were necessary for a change in both the community action and access to help. They called for clear laws and regulations to protect children from substance use, as well as increased priority and investment in prevention and handling of this issue. They looked to Kenya, which had been successful in implementing restrictions on access to alcohol by reducing the opening hours of bars. While government action was considered necessary, there was considerable hesitation about its ability and commitment to act, and community involvement in the process was considered necessary.

#### ‘We don’t have clear laws’

The participants explained that a considerable challenge was the weak protection of children from substance use in the current laws and regulations in Uganda: “*It has always been like this because we don’t have clear laws which control alcoholism in our constitution.” (KII 8, mental health activist).* The participants suggested restrictions on the opening hours of bars, stronger enforcement of the age limit and regulation of brewing. They explained that in the current situation, the responsibility was put on each community member to make individual decisions to do what was best for the child. They called for government leadership and action in the form of clear laws to strengthen the community mandate to act, and to act coherently:

“*A voice from the government or higher authorities should come down here to the local council, who can call for meetings so that the community can sit, and they go through what is happening to the children, and then come out with a law.*”* (FGD 5, younger women).*

The participants explained that putting the responsibility to act on behalf of children on the individual community member, without supporting laws, could lead to difficult dilemmas. This ranged from the risk of compromising community relationships by unwanted interference to the more explicit dilemma of earning money on selling alcohol to children:

“*I need money to survive. If I don’t sell to those young children, but other friends are selling, you find there is nothing you have done. Unless you agree all that ‘we are not supposed to do this, we are not supposed to do this’, but when you are not together [agreeing], you cannot do it. You can’t.*”* (KII 12, alcohol distributor).*

Although participants, including bar owners, agreed that children should not be able to buy alcohol, they recognised the challenge for the individual alcohol seller to self-impose this restriction when it meant losing income and achieving little unless everyone changed practice through government regulations. However, while the government was perceived necessary for action, the belief in this materialising was tepid.

#### ‘It may never be addressed’

The participants’ belief in the government’s ability and commitment to act was limited. The local council was considered key when it came to the development and implementation of policy and regulations and were perceived more likely to have an impact: *“If it is not addressed right from the grassroot level, then trust me, it may never be addressed.” (KII 18, lawyer).* There was variation in the extent to which participants believed the government was informed or dedicated to addressing early childhood substance use. Some believed the government did not know, while others believed they did not care to act unless it involved personal gains:

“*The government has not done so much. Most of them look at their salary, and that is it. They don’t come down to the grassroot to find out about the problems. […] I know you might make recommendations after your research, but they are not going to do anything. The government has a very big part to be blamed, they just put things on paper, but they don’t put them in implementation. They have to wake up.*”* (KII 1, primary school teacher).*

Some participants explained that there was a complex interplay between the government, the alcohol industry and election campaigns, where liquor samples would be distributed for free. Some participants believed that corruption and self-interest could be a factor for politicians and the non-governmental organisations (NGO), raising concerns that they were making a profit of the problem, and solving it would undermine their existence:

“*I'm sorry to say, people make it look like rocket science to end it [children on the street]. It is possible to end it, but it seems that if you are having a problem, it gets money, you don't want to get rid of it. […] I knew this guy from this organisation who was riding a bicycle before he started the organisation, soon we saw him in a car and soon he was driving a range rover sport, and I ask myself, if all this money is still coming here, and you can build these facilities, how come we are getting more street children?*”* (KII 26, youth worker).*

The government and NGOs were the ones with perceived real power to act but were not believed to channel this power into actions that would give community members a mandate to address childhood substance use, or the nescessary resources for institutions to manage it.

In summary, all three themes on addressing, managing, and acting for childhood substance use were permeated by a sense of powerlessness due to the magnitude and complexity of the issue. The participants expressed a deep concern for childhood substance use but felt incapacitated in tackling it unless everyone was part of it on all levels, including the community, institutions, and the government.

## Discussion

In this study, we explored community members’ perceptions on how childhood substance use was addressed in the communities, managed in relevant institutions, and acted upon by the government. The participants expressed concern for childhood substance and alcohol use but perceived the problem to be too complex for any individual to tackle alone. They explained that the community members’ agency was hampered by a lack of leadership in addressing the issue, changes in traditional collective child-rearing practices and restrictions related to children’s rights policies. The participants considered schools and correctional facilities, such as remand homes, to be appropriate institutions for intervention, while the health system was mostly sought if the substance use had led to adverse consequences. These institutions’ opportunity to manage early childhood substance use was, however, hampered by inadequate human and financial resources. Finally, the participants called for stronger policies and laws from the government to limit the access and use of substances but had little trust in their ability to act. We will now discuss these findings considering elements from the WHO Commission on Social Determinants of Health framework on social determinants related to social cohesion, community agency, power, and governance [22].

### Social cohesion and the power to act

All three themes were permeated by a perceived lack of power and agency in handling childhood substance use. This notion spanned community members’ hesitation to intervene with other’s children, the police officer’s inability to tackle the sheer magnitude of children using substances and the alcohol seller who alone could not stop access of alcohol to children. The WHO emphasises that social determinants of health include social cohesion (the social integration, mutual trust and belonging to a community), the social capital (social cohesion as a resource that facilitates collective action for mutual benefit), and the power to act (the mandate and opportunity for social participation in shaping their community). Further, it states that it “*requires understanding how power operates in multiple dimensions of economic, social and political relationships”* [22]. These factors are recognised in our findings where the social relationships hampered community discussion and intervention; economic relationships made it difficult to access care and limit sales of alcohol to children; and political relationships and the perceived absence of political commitment hindered action on all levels.

In our findings we observe a perceived powerlessness and inability to act on a community, institutional, and government level. Social cohesion, collective agency and power have been explored in political and psychological theory, as well as in empirical studies. The political philosopher Arendt explained that; *“power corresponds to the human ability not just to act, but to act in concert. Power is never the property of an individual; it belongs to a group and remains in existence only so long as the group keeps together”* [22, 26]*.* In psychology, Bandura’s theory on ‘self-efficacy’ [27] and ‘collective efficacy’ [28] has been central in understanding agency in the face of challenges. These factors can have important implications for childhood substance use, as empirical studies show that community inaction is associated with increased substance use among youth. A systematic review of community level social factors and alcohol use found that, overall, community disorder and crime were risk factors, while safety and social capital (community attachment and support, community participation) were protective [29]. We note that all 48 studies included in the review were from high income settings, except one from Bolivia. However, studies from South Africa have shown similar results as presented in the systematic review, demonstrating that neighbourhood belonging and encouragement (community affirmation) was protective for intake of brew by adolescents [30]. Risk factors for increased alcohol intake among youth included neighbourhood crime and antisocial behaviour among neighbourhood adults (neighbourhood disorganisation) [30] and living in a community where it was perceived to be unlikely that the police or neighbourhood members would respond to youth drinking [31]. Among adult men, identified protective factors for heavy drinking included higher level of collective efficacy, measured by perceived informal social control and social cohesion [32]. Interestingly, informal social control was assessed by asking how likely it would be for your neighbour to intervene if a child was observed skipping school or being disrespectful [32], tapping into the concerns raised by the participants about the loss of mandate to intervene when observing children that use substances. We have not identified studies that have investigated these community mechanisms in relation to children below age 10, but our findings can imply that similar factors are relevant in this age group in Uganda.

Community action and empowerment is an important entry point for prevention of and intervention for early substance use [3]. Studies have suggested that a community-based approach is even more important in the African context [30] because of a long-standing collectivist orientation [5, 30]. In addition to the power and collective efficacy yielded by social cohesion and social capital, this collectivist sense of group belonging has been suggested to have an overall protective effect on mental illness [5]. Therefore, it is worth paying attention to our finding of a perceived disintegration of this collectivist social fabric and its implications for community members to identify, intervene and support children and parents of children that drink alcohol or use other substances.

### Governance and policy implications

The participants in our study had several suggestions for policy improvements to protect children from alcohol and other substances, including restrictions on days and hours for sale and stronger enforcement of the age limit. These policy suggestions are overlapping with the WHO Global Strategy to Reduce the Harmful Use of Alcohol [3], which notes a discrepancy between the high impact of alcohol to public health and low priority among decision-makers [3]. Unfortunately, the participants’ concern for a lack of priority of these policy measures was confirmed in 2017, when the WHO published a report on the “Progress in implementation of the WHO global strategy to reduce the harmful use of alcohol since 2010”, finding that no low-income country had increased their resource allocation for implementing alcohol policies [33]. The participants were concerned that this lack of priority may be affected by stakeholders’ self-interests and influence by the alcohol industry. Concerns about the exploitation of weak alcohol policies in Africa has previously been raised, where the industry has increased their production and promotion in the region and lobby to keep the market unregulated [34]. Despite this, examples from South Africa and Botswana show that when there is a political will, increasing taxes and restricting the availability of alcohol can help reduce intake, including among youth [2]. This political will has also been observed in Uganda’s effort to ban small sachets of liquor due to the harm they pose to children and adults, but their efforts are receiving push-backs from the industry [35]. That said, brewing is the livelihood for many, and in the effort to regulate alcohol access and output it is important to simultaneously stimulate alternative sources of income [3].

Another governance issue raised by the participants was the inadequate financial and human resources in the education, police, and health sector. The WHO highlights the importance of health service response [3], and the Ugandan Ministry of Health acknowledges the need *“to build capacity, improve access and availability of comprehensive Mental Neurological and Substance use services for care and treatment of children and adolescents […]”* [36]. However, given the stated resource constraints in responding to childhood substance use and the detrimental economic and social effects throughout the life-course, efforts to prevent early initiation of substance use are crucial. This is emphasised in the WHO Global Strategy to Reduce the Harmful Use of Alcohol [3], which outlines that the most cost-effective strategies for reducing the harmful effects of alcohol, termed “best buys” [3], include preventative measures that regulate alcohol availability, i.e., minimum legal drinking age and restricting hours, days, and places of sale, restricting and enforcing bans on alcohol marketing, and regulating prices and taxes [3]. Further, the participants explained that the health sector was not even perceived relevant to approach for substance use issues. These context specific factors are important to keep in mind and address when designing and planning interventions [5]. Further, according to the WHO, a multidisciplinary and multisectoral approach is key as *“policies to reduce the harmful use of alcohol must reach beyond the health sector, and appropriately engage such sectors as development, transport, justice, social welfare, fiscal policy, trade, agriculture, consumer policy, education and employment, as well as civil society and economic operators”* [3]. Our findings suggest that focusing efforts on schools and disseminating alternative disciplining strategies that are in line with children’s rights may be relevant in Uganda. Further, it is necessary to address how children’s rights, that are meant to protect children, are now perceived as a barrier for community members to act on their behalf. While banning corporal punishment undoubtedly has positive effects [37], shortcomings in implementation has left parents and other adults unsure about alternative ways for correcting unwanted behaviour in children [38]. In an effort to remove harmful practices, we may leave a vacuum unless we provide beneficial alternatives. This point parallels to one made in a previous paper, where the same participants warned against removing the substances that children used for coping with cold, hunger and traumatic experiences without providing alternatives [18].

### Strengths and limitations

This study provides important insights into community experiences and perceptions regarding how childhood substance use is addressed, managed, and acted upon in Mbale District, Uganda. With a relatively large and diverse sample of participants, we have explored the viewpoints of a broad representation of the community. However, a major limitation is the missing experiences of children who are using substances and their parents, and how they perceive the support from the community, institutions, and government. These insights would be invaluable to fully capture the lived experiences of these families and to inform how to best support them. Methodological strengths include triangulation of methods (KIIs and FGDs), and participant feedback on findings. Furthermore, our research assistants were bilingual and translated the transcripts in pair reaching consensus, but we appreciate the limitations in analysing translated transcripts [25]. Moreover, we note that, due to capacity issues, the data was collected in 2016 and analysed from 2019–2021. The time passed may have included changes that are not accounted for, including the detrimental consequences of the Covid-19 pandemic. However, we have not observed other substantial changes since the time of data collection that we believe would have impacted our findings or conclusions considerably.

## Conclusion

In this paper we have explored community members’ experiences and perceptions regarding how childhood substance and alcohol use was addressed, managed, and acted upon at the community, institutional and government level. We have learnt that while the practice was common knowledge, and of deep concern, it was not addressed at the community level. A lack of leadership and social cohesion hampered collective community action. Relevant institutions for seeking help were underfunded and unable to manage the problem. There were ample suggestions of evidence-based policy measures, but these were complicated by power relations between stakeholders and the industry. Context-specific perceptions about the irrelevance of the health system for managing substance use, the perceived importance of schools, and experiences of children’s rights as a barrier to helping children underlined the complex context that needs to be considered when suggesting and planning interventions. A multidisciplinary and multisectoral approach, tackling barriers and drawing on opportunities in the community, relevant institutions for seeking help, and the government, is important. We have established that the community knows and cares about the issue, and welcomes discussion and action on childhood substance use. The community is instrumental for tackling childhood substance use, and their insights are invaluable for informing policy and implementing future interventions. We reiterate the need for research focusing on the lived experiences of children using substances and their parents.

## Supplementary Information

**Additional file 1. **Case vignette for the focus group discussion.

## Data Availability

The University of Bergen and Makerere University have shared intellectual property rights to the data. The datasets used and analysed during the current study are available from the corresponding author on reasonable request.

## Abbreviations

APC: Alcohol per capita consumption
FGD: Focus group discussion
KII: Key informant interview
LMIC: Low- and middle-income countries
NGO: Non-governmental organisation
WHO: World Health Organization
