# Supplementary material for: ‘There is nowhere to take the child’: a qualitative study of community members’ views on managing early childhood substance use in Mbale, Uganda
Source: BMC Public Health. 2022 Jun 15;22:1192. doi: 10.1186/s12889-022-13548-4 (PMC9198618; doi:10.1186/s12889-022-13548-4)
Supplement: Supplementary file 1 — Additional file 1. Case vignette for the focus group discussion. [file 12889_2022_13548_MOESM1_ESM.docx]

*Case vignette for the focus group discussion*

There is a party in the village as one of the boys have graduated from a bachelor’s degree. He is from a wealthy family that is hosting a big celebration. After the ceremonies there is a party with a lot of dancing and alcohol is being served. Some of the boys of nine years old are sharing a bag of waragi, while a girl of six years is drinking some brew. The day after the girl gets some alcoholic brew for lunch that the mother is making and selling. She likes the brew and it makes her sleep better. After a while she can tolerate more and drinks some before school to get rid of her head aches.

Can you discuss this story a bit for me, and discuss if it is something that can happen in this community?
